# Identification of Differentially Abundant Proteins of *Edwardsiella ictaluri* during Iron Restriction

**DOI:** 10.1371/journal.pone.0132504

**Published:** 2015-07-13

**Authors:** Pradeep R. Dumpala, Brian C. Peterson, Mark L. Lawrence, Attila Karsi

**Affiliations:** 1 The Rogosin Institute, Xenia Division, Xenia, Ohio, United States of America; 2 USDA ARS Warmwater Aquaculture Research Unit, Thad Cochran National Warmwater Aquaculture Center, Stoneville, Mississippi, United States of America; 3 Department of Basic Sciences, College of Veterinary Medicine, Mississippi State University, Mississippi State, Mississippi, United States of America; Henan Agricultural Univerisity, CHINA

## Abstract

*Edwardsiella ictaluri* is a Gram-negative facultative anaerobe intracellular bacterium that causes enteric septicemia in channel catfish. Iron is an essential inorganic nutrient of bacteria and is crucial for bacterial invasion. Reduced availability of iron by the host may cause significant stress for bacterial pathogens and is considered a signal that leads to significant alteration in virulence gene expression. However, the precise effect of iron-restriction on *E*. *ictaluri* protein abundance is unknown. The purpose of this study was to identify differentially abundant proteins of *E*. *ictaluri* during *in vitro* iron-restricted conditions. We applied two-dimensional difference in gel electrophoresis (2D-DIGE) for determining differentially abundant proteins and matrix-assisted laser desorption/ionization time-of-flight mass spectrometry (MALDI TOF/TOF MS) for protein identification. Gene ontology and pathway-based functional modeling of differentially abundant proteins was also conducted. A total of 50 unique differentially abundant proteins at a minimum of 2-fold (p ≤ 0.05) difference in abundance due to iron-restriction were detected. The numbers of up- and down-regulated proteins were 37 and 13, respectively. We noted several proteins, including EsrB, LamB, MalM, MalE, FdaA, and TonB-dependent heme/hemoglobin receptor family proteins responded to iron restriction in *E*. *ictaluri*.

## Introduction

*Edwardsiella ictaluri* causes enteric septicemia in catfish (ESC), which is one of the most prevalent bacterial diseases affecting farm-raised catfish in the United States [1]. ESC can occur either as an acute or a chronic disease in catfish, and it is capable of causing high mortalities [2–4]. Previous studies have identified potential virulence factors of *E*. *ictaluri*, including extracellular capsular polysaccharide [5], lipopolysaccharide (LPS) [6–11], outer membrane proteins (OMP) [11–15], hemolysins [16], and chondroitinase [5, 17, 18]. Previous research has also shown that *E*. *ictaluri* is able to survive and replicate inside catfish neutrophils and macrophages [2, 4, 5, 19, 20].

Iron is an essential micro element for almost all living organisms and is involved in various metabolic processes like sugar, protein, energy, and DNA metabolism, growth, and response to oxidative stress [21]. Reduced availability of iron may cause significant stress for bacterial pathogens and is considered a signal that leads to significant changes in gene expression [22]

Vertebrate hosts tend to chelate free iron using high affinity proteins like ferritin, transferrin, and heme proteins, which restricts iron availability for bacteria [23, 24]. This innate mechanism of iron-restriction by the host is an important host defense mechanism against bacterial infection [25, 26]. In turn, low levels of iron in the environment often trigger virulence factor expression in pathogens [27]. In many Gram-negative bacteria, iron associates with ferric uptake regulator (Fur) to regulate expression of virulence genes [28]. Based on this phenomenon, a significant number of potential virulence genes have been identified in *E*. *coli* [29, 30], *E*. *ictaluri* [31], *Shigella dysenteriae* [32], *Vibrio cholera* [33–36], *Neisseria meningitidis* [37], and *Pseudomonas aeruginosa* [38–40].

High throughput proteomics methods have the potential to accelerate discovery of virulence determinants of *E*. *ictaluri*. Previously, we analyzed and annotated the sub-proteome of *E*. *ictaluri* strain 93–146 [41]. We now report how the *E*. *ictaluri* sub-proteome responds when grown under iron-restricted conditions. This information has the potential to elucidate mechanisms of ESC pathogenesis at the molecular level.

## Materials and Methods

### Iron-restricted growth and total protein extraction

*E*. *ictaluri* strain 93–146 [42] was grown on brain heart infusion (BHI) broth or agar medium. Chelating agent 2,2′-dipyridyl (Sigma, St. Louis, MO.) at a final concentration of 100 mM was used to sequester iron from the medium [31, 43–45]. Triplicate control (grown in BHI broth) and treatment (grown in iron-restricted BHI) cultures of *E*. *ictaluri* were harvested at mid-exponential phase (OD_600_ 0.6) by centrifugation at 2,800 x g for 15 min at 30°C.

Six bacterial pellets (three control and three treatment) were washed three times using standard cell wash buffer (10 mM TRIS hydrochloride (Tris-HCl) and 5 mM magnesium acetate) at 30°C and were suspended in 750 uL of urea-CHAPS buffer (8 M urea, 30 mM Tris-HCl, 4% 3-[(3-Cholamidopropyl)dimethylammonio]-1-propanesulfonate (CHAPS), 8 mM phenylmethanesulfonyl fluoride pH 8.0). Bacteria were lysed on ice by applying ten intermittent pulses of 10 s with a sonicator, and cellular debris was removed by centrifugation at 4°C at 20,817 x g for 5 min.

Proteins from supernatant were precipitated by trichloroacetic acid/acetone, and the resultant protein pellets were suspended in urea-CHAPS buffer. The pH of the lysates was adjusted to 8.5 using 50 mM sodium hydroxide. Protein concentrations were estimated using a 2-D Quant Kit (GE Healthcare, Piscataway, NJ) following the manufacturer’s instructions.

### Labeling of proteins

Protein samples were labeled using a CyDye difference in-gel electrophoresis (DIGE) Fluor minimal labeling kit (GE Healthcare) according to the manufacturer’s manual. Briefly, 50 μg of protein from an internal standard (equal mixture (8.33 μg) of all 6 samples), control, and treatment were mixed with 400 pmol of Cy2, Cy3, or Cy5 dyes, respectively, and protein-dye mixtures were incubated on ice in the dark for 30 min. Labeling reaction was terminated by adding 1 μl 10 mM lysine, mixing well, and incubating samples in the dark for 10 min.

### Protein separation using two-dimensional gel electrophoresis (2-DE)

For isoelectric focusing (IEF), precast 17 cm pH 3–10 NL immobilized pH gradient (IPG) strips (Bio-Rad, Hercules, CA) were used. Each of the labeled protein samples from control, treatment, and internal standard were combined with rehydration buffer containing 7 M urea, 2 M thio urea, 4% CHAPS, 1:50 carrier ampholyte, and 2% DTT. Mixed samples were loaded onto each IPG strip for in-gel rehydration. IEF was performed in a Protean IEF cell (Bio-Rad, Hercules, CA) in the dark at 23°C, 500 V for 15 min; linear ramp to 10,000 V for 3 h; and 10,000 V until a total of 70,000 Vh was reached. After IEF, IPG strips were equilibrated in 6 M urea, 30% glycerol, 50 mM Tris-HCl, 2% sodium dodecyl sulfate (SDS), 2% dithiothreitol (DTT), at pH 8.8, and with a trace of bromophenol blue for 15–20 min followed by equilibration containing 2.5% iodoacetamide (IAA) instead of 2% DTT for 15–20 min. Once equilibrated, strips were transferred onto 12% SDS-polyacrylamide gel electrophoresis gels (Jule Inc., Milford, CT) and sealed with 0.5% agarose in electrophoresis buffer. Electrophoresis was performed using a PROTEAN II XL system (Bio-Rad) at a constant current of 10 mA/gel for the first 15 min followed by 24 mA/gel at 20°C until the dye front reach the lower end of the gel.

### Analysis of 2-D DIGE gel images

After electrophoresis, DIGE gels were scanned using a Typhoon 9410 imager (GE Healthcare). Excitation and emission filters used for each dye were as follows: Cy2 (488 nm/520 nm), Cy3 (532 nm/580 nm), and Cy5 (633 nm/670 nm). Acquired images were analyzed using DeCyder 5.0 software (GE Healthcare). Briefly, spots were detected using the differential in-gel analysis (DIA) module. Spot matching between gels and statistical analysis of protein-abundance changes were conducted using the biological variation analysis (BVA) module. Among the three replicates, the gel with the highest number of spots was assigned as the master gel. All the spots that were matched automatically were also manually compared among all 3 replicate gels to minimize false spot matching. Statistical significance was calculated using the Student’s t-test with applied false discovery rate and a significance threshold of *p* < 0.05. Only spots showing at least 2-fold change in spot intensity and were consistent in all three replicate gels were considered as differentially abundant and chosen for protein identification.

### Preparative gel electrophoresis and protein identification

Preparative 2-DE gels were prepared exactly as described above, except that the IPG strips were loaded with 500 μg of protein. Resultant gels were stained using Deep Purple Total Protein Stain (GE Healthcare) according to the manufacturer’s protocols. Briefly, gels were fixed overnight in 15% v/v ethanol and 1% w/v citric acid followed by staining for 1 h in 1:200 parts of Deep Purple and 100 mM sodium borate solution at pH 10.5. Gels were then washed for 30 min with 15% v/v ethanol in water, acidified for 30 min using a solution containing 15% v/v ethanol and 1% w/v citric acid. Stained gels were scanned using a Typhoon 9410 imager using a 532 nm laser and a 610 nm BP30 emission filter. In-gel trypsin digestion and MALDI peptide mass fingerprinting (PMF) was performed as previously described [46] with slight modifications (mass tolerance value was 150 ppm and *E*. *ictaluri* protein database was used).

### Functional modeling of differentially abundant proteins

We used gene ontology (GO) resources GORetriever and GOanna (available at AgBase) [47] for obtaining biological process and molecular functional annotations of differentially abundant proteins. Using GORetriever, we obtained all existing GO annotations for proteins. Proteins with no existing GO annotations but with a sequence similarity of >80% with presumptive orthologs were annotated using Goanna. Obtained GO biological process and molecular function annotations were manually summarized to more generalized categories based on the ancestor chart for GO terms at QuickGO [48]. The subcellular locations of differentially abundant proteins were predicted using PSORTb v3.0.0 [49]. To gain insight into various biological pathways that were significantly represented by our differentially abundant proteins, we used Pathway Studio 6.0 (Ariadne, Rockville, MD) as previously reported [41]. In addition, “build pathway” function was used to build a biological interactions network of up- and down-regulated proteins.

## Results

### Identification of differentially abundant proteins

**Fig 1:**
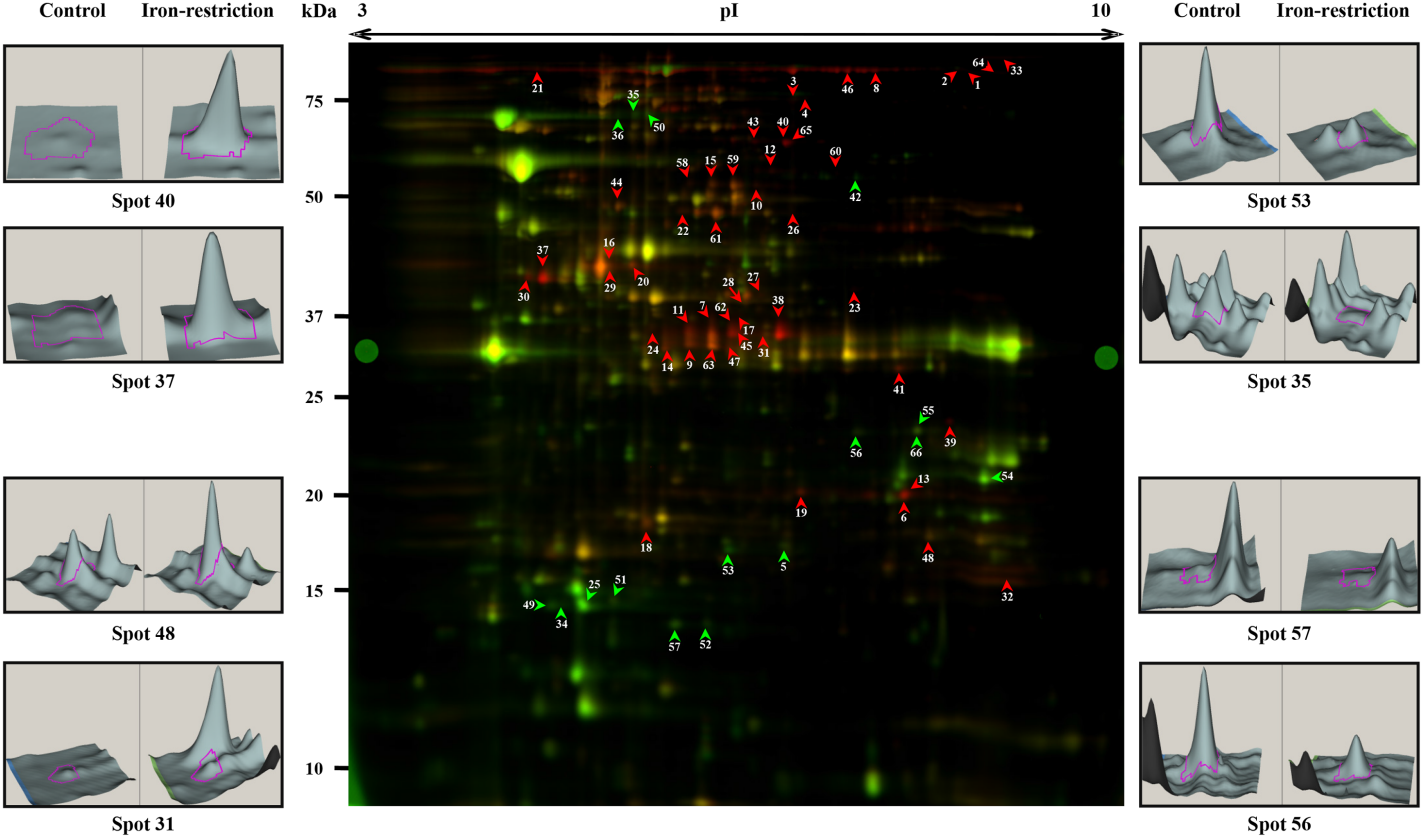
Fluorescent difference gel electrophoresis (2-D DIGE) of *Edwardsiella ictaluri* grown in iron-rich and iron-restricted conditions. Fifty μg of soluble protein from *E*. *ictaluri* grown in regular brain heart infusion (BHI) media was labeled with Cy3, grown in BHI with chelator 2, 2’-dipyridyl was labeled with Cy5, and the pooled internal standard labelled with Cy2. Spots shown in red and green arrow head are up- and down-regulated (≥2 fold), respectively. 3D images of 2 spots with maximum and minimum fold up-regulated proteins were shown on left top and bottom corners of gel image, respectively. 3D images of 2 spots with maximum and minimum fold down-regulated proteins were shown on right top and bottom corners of gel image, respectively.

The DIGE analysis detected approximately 2,200 spots in each replicate, and after automatic matching and manual verification of each spot, only those spots that were matched in all 3 replicate gels were subjected to statistical analysis. Analysis of these spots revealed that 131 spots (92 up- and 39 down-regulated) were differentially abundant with a minimum of 2-fold, *p* < 0.05 in iron-restricted conditions compared to bacteria grown in regular BHI media. Among the 131 spots, 71 spots (54 up- and 17 down-regulated) matched to a preparative gel were cut for mass spectrometric analysis, and 65 (91.54%) positive identifications with confidence intervals > 99% were identified (Fig 1). Fifteen proteins were represented in more than one spot, probably due to migration of abundant proteins to more than one spot or post-translational modifications and processing. In conclusion, we were able to determine 50 (37 up- and 13 down-regulated) unique differentially abundant *E*. *ictaluri* proteins under *in vitro* iron-restricted conditions (Table 1). Notable among these were EsrB, LamB, MalM, MalE, Fda, AspA, DsbA, OmpA, OppA, and TonB-dependent heme/hemoglobin receptor family protein.

**Table 1:** Differentially regulated proteins of *Edwardsiella ictaluri* in response to *in vitro* iron-restriction.

Process/GI number	Protein number	Spot ID	Fold difference	CI%	Protein name	Protein MW/PI	Pep. count	Protein score	Gene name
**Alcohol metabolic process**									
238919566	1	194/195	3.82/2.68	100	Aldehyde-alcohol dehydrogenase 2	95992.7/6.41	28	413	NT01EI_1665
**Biosynthetic process**									
238919324	2	350/346	2.89/2.49	100	Bifunctional polymyxin resistance protein ArnA, putative	73954/5.67	26	264	arnA/NT01EI_1415
238920260	3	1972	-4.14	100	3-oxoacyl-[acyl-carrier-protein] reductase, putative	25567.1/5.95	9	244	NT01EI_2369
**Carbohydrate metabolic process**									
238921292	4	1811	5.48	100	N-acetylmuramoyl-L-alanine amidase AmiD	28647.3/6.6	11	220	NT01EI_3435
238920353	5	182	3.63	100	Formate acetyltransferase, putative	85051.6/5.65	22	154	NT01EI_2463
238921224	6	1347/1352/1316	3.03	100	Fructose-bisphosphate aldolase, putative	39129.7/5.65	14	382	fba or fda/NT01EI_3367
238918053	7	1301	3.55	99.99	ADP-glyceromanno-heptose 6-epimerase, putative	34791/5.29	9	77	hldD/NT01EI_0072
238918174	8	715	2.45	100	Glucose-6-phosphate isomerase	61392.9/6.06	19	243	pgi/NT01EI_0210
238920733	9	1809	3.05	100	Hypothetical protein NT01EI_2846	28196.7/6.56	18	454	gpmA/NT01EI_2846
238921491	10	699/690	3.16/3.24	100	Phosphoenolpyruvate carboxykinase (ATP)	59171.9/5.77	28	571	pckA/NT01EI_3643
**Nucleoside/nucleotide metabolic process**									
238918513	11	1034	2.84	100	Thymidine phosphorylase, putative	46793.9/5.33	14	139	NT01EI_0563
238918595	12	1236	2.33	100	Hypothetical protein NT01EI_0651	38512.4/8.34	12	209	NT01EI_0651
238918515	13	1888	2.17	100	Purine nucleoside phosphorylase, putative	25636.8/5.4	11	292	deoD/NT01EI_0565
238918109	14	1801	3.33	100	Uridine phosphorylase, putative	27335.9/6.07	13	645	NT01EI_0133
238918514	15	1051	2.17	100	Phosphopentomutase, putative	44429.2/5.33	20	341	deoB/NT01EI_0564
**Oxidation reduction**									
238918700	16	147	2.46	100	Pyruvate dehydrogenase; acetyl-transferring, homodimeric type, putative	99427.8/5.55	20	169	NT01EI_0758
238918702	17	819/833	2.51	100	Dihydrolipoyl dehydrogenase, putative	50803.5/5.64	19	397	NT01EI_0760
238919229	18	1144	4.49	100	Udp-glucose 6-dehydrogenase	43359.6/6.09	11	113	NT01EI_1312
238918818	19	1288	3.08	100	1,3-propanediol dehydrogenase	40188.1/5.45	15	467	NT01EI_0882
238920005	20	2096	-3.67	100	Superoxide dismutase	21120.4/5.26	5	364	Sod_Fe/NT01EI_2109
**Phosphorylation**									
238921741	21	713	3.68	100	ATP synthase subunit alpha/ AltName: F-ATPase subunit alpha	55190.7/5.59	24	491	atpA/NT01EI_3910
238920582	22	1171	2.1	100	Acetate kinase, putative	43096/5.9	17	517	NT01EI_2694
238920730	23	1243	2.45	100	Galactokinase, putative	41138.9/5.83	18	411	NT01EI_2843
**Translation**									
238921444	24	1082	2.28	100	Elongation factor Tu	43262.2/5.15	19	672	NT01EI_3596
238918136	25	1095	3.71	100	Translation elongation factor Tu, putative	43262.2/5.15	23	866	NT01EI_0167
238919786	26	1289	2.02	100	Phenylalanyl-tRNA synthetase, alpha subunit, putative	36890.7/5.9	25	581	pheS/NT01EI_1890
238921441	27	2020	2.47	100	50S ribosomal protein L4	22068.8/9.72	7	198	rplD/NT01EI_3593
238918424	28	102	6.95	100	Translation initiation factor IF-2, putative	98155.7/5.72	21	158	infB/NT01EI_0467
238921430	29	2095	-2.95	100	RecName: Full = 50S ribosomal protein L5	20333.7/9.59	12	248	rplE/NT01EI_3582
238917996	30	428/406	-2.61/-4.78	100	Glycyl-tRNA synthetase, beta subunit, putative	75997.9/5.35	37	633	glyS/NT01EI_0014
**Transport**									
238918184	31	1099	8.77	100	Maltoporin	46962.3/5.18	18	576	lamB/NT01EI_0220
238918180	32	1314/1303	8.73	100	Bacterial extracellular solute-binding protein, putative	43474.5/6.48	24	494	malE/NT01EI_0216
238918185	33	1570	4.86	100	Maltose operon periplasmic protein	31714.5/8.77	8	121	malM/NT01EI_0221
238919805	34	551/541	17.95	100	TonB-dependent heme/hemoglobin receptor family protein	72860.4/6.13	33	457	chuA/NT01EI_1909
238920966	35	1442	2.74	100	ABC transporter, substrate binding protein	37823.6/7.79	14	352	NT01EI_3096
238919569	36	675	-2.82	100	Periplasmic oligopeptide-binding protein	61472.2/6.82	12	136	oppA/NT01EI_1668
**Tricarboxylic acid cycle**									
238920751	37	540	6.3	100	Succinate dehydrogenase, flavoprotein subunit, putative	64419.1/5.94	29	500	sdhA/NT01EI_2870
238918339	38	795	3.25	100	Aspartate ammonia-lyase, putative	52454.8/5.33	17	436	aspA/NT01EI_0377
**Others**									
238921325	39	1353	2.1	100	Glycerophosphoryl diester phosphodiesterase	40878.5/6.01	17	303	NT01EI_3469
238920583	40	188	4.22	100	Phosphate acetyltransferase	76925/5.46	21	221	NT01EI_2695
238918772	41	1364	2.48	100	Methionine aminopeptidase, type I, putative	29710.1/5.63	12	298	NT01EI_0835
238918900	42	1935	2	100	Hypothetical protein NT01EI_0965	23510.7/6.92	14	359	esrB/NT01EI_0965
238919128	43	2094	-3.03	100	Glycine cleavage system transcriptional repressor	20825.4/5.03	7	82	gcvR/NT01EI_1199
238921227	44	407	-2.51	100	Transketolase 1 (TK 1)	72356/5.66	19	197	tktA/NT01EI_3370
238921714	45	2099	-3.35	99.99	Thiol:disulfide interchange protein DsbA	22948.7/5.79	5	79	dsbA/NT01EI_3876
238921092	46	2148	-2.74	100	Hypothetical protein NT01EI_3227	18959.4/5.6	9	330	luxS/NT01EI_3227
238919302	47	1968	-4.79	100	Outer membrane protein A	38075.3/8.79	12	200	ompA/NT01EI_1392
238919598	48	1868	-2.68	100	Hypothetical protein NT01EI_1697	27900.5/8.98	15	307	NT01EI_1697
238920203	49	1596/1598	-2.78/-2.34	100	Hypothetical protein NT01EI_2312	31984.4/6.92	21	656	NT01EI_2312
238920625	50	2159	-2.33	100	Hypothetical protein NT01EI_2737	19363.8/5.29	9	123	eip20/NT01EI_2737

### Functional modeling of differentially abundant proteins

**Fig 2:**
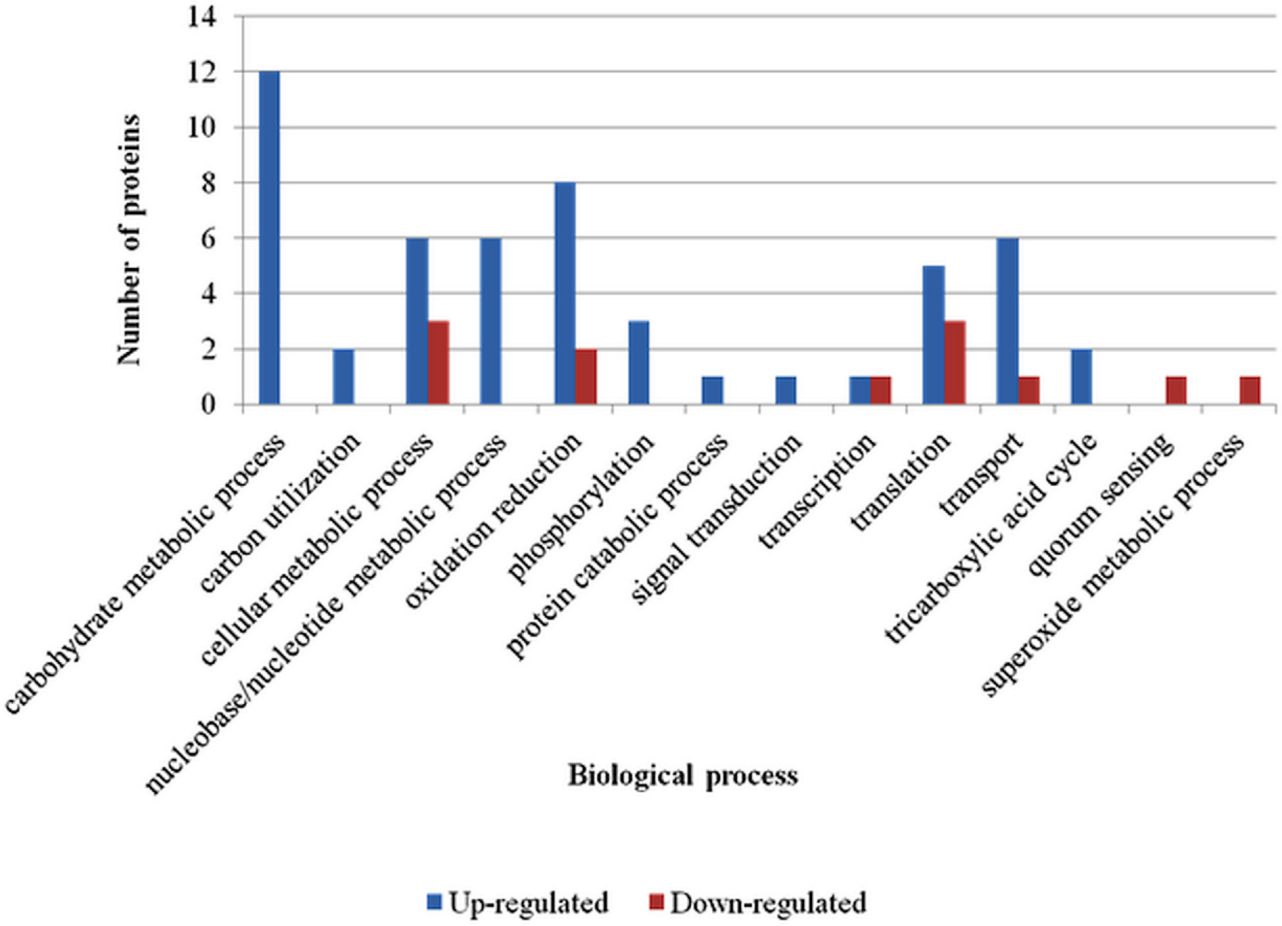
Biological process gene ontology (GO) Slim of differentially abundant proteins of *Edwardsiella ictaluri* grown in *in vitro* iron restriction condition. All biological process GO annotations of up- and down-regulated proteins were summarized to more generalized GO categories based on ancestor chart for GO terms at QuickGO. Number of proteins involved in various generalized GO biological process categories was represented.

**Fig 3:**
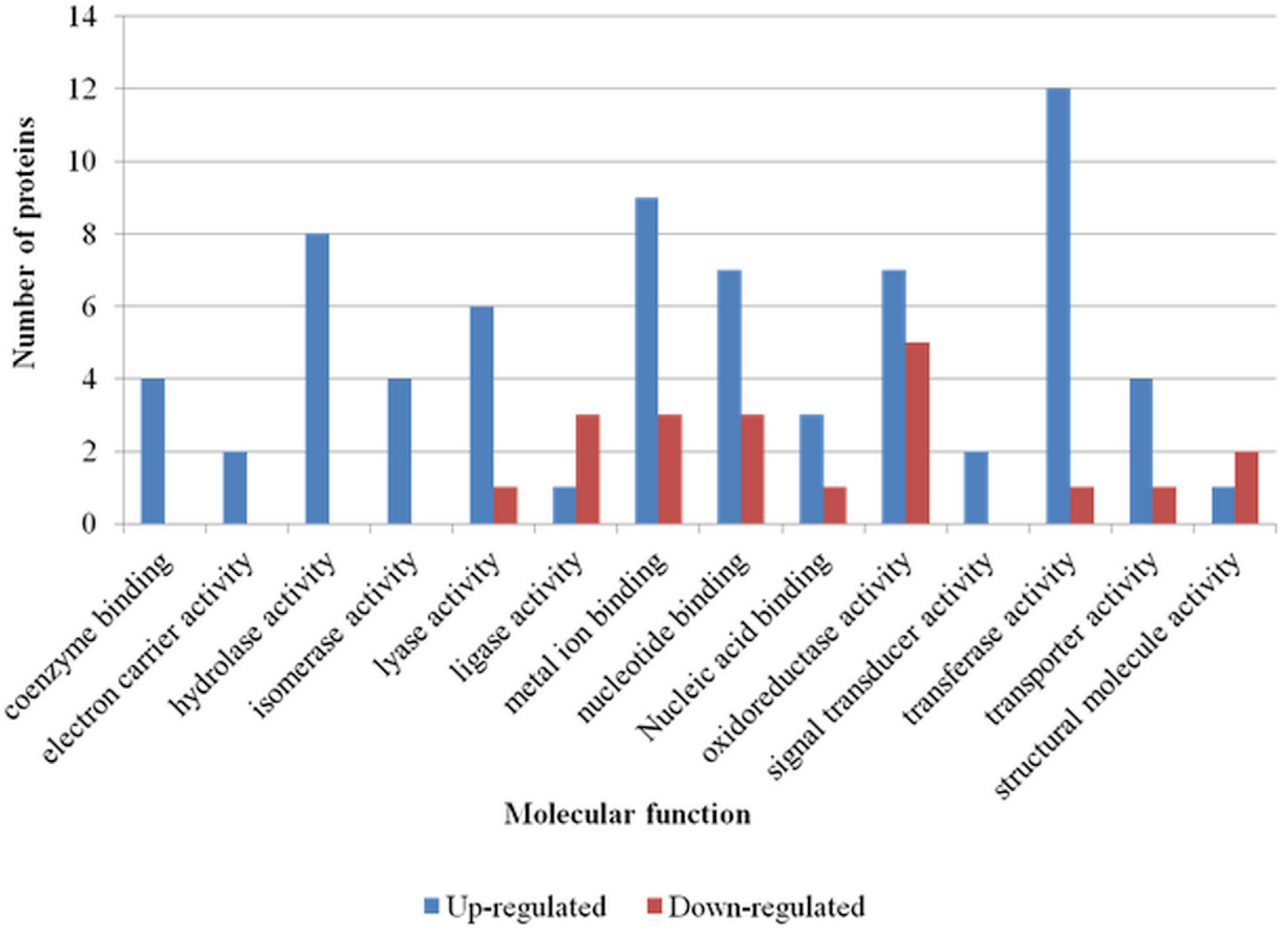
Molecular function gene ontology (GO) Slim of differentially abundant proteins of *Edwardsiella ictaluri* grown in *in vitro* iron-restriction condition. All molecular functional GO annotations of up- and down-regulated proteins were summarized to more generalized GO categories based on ancestor chart for GO terms at QuickGO. Number of proteins involved in various generalized GO molecular functional categories was shown.

GO annotation of the 50 unique differentially abundant proteins and manual slimming based on GO terms resulted in 14 biological process (Fig 2) and 14 molecular function (Fig 3) categories. Up-regulated proteins were represented in 12 biological processes, whereas down-regulated proteins were represented only in 7 biological processes. The top three biological process categories represented by higher numbers of up-regulated proteins were carbohydrate metabolic process, oxidation reduction, and cellular metabolic process. Two of these categories (cellular metabolic process and oxidation reduction) were also among the top three biological processes involving down-regulated proteins. Interestingly, carbohydrate metabolic processes, which included the highest number of up-regulated proteins, did not include any down-regulated proteins.

Up-regulated proteins were represented in all 14 molecular functional categories, whereas down-regulated proteins were represented only in 9 molecular functional categories. In the molecular function grouping, transferase activity, metal ion binding, and hydrolase activity were the top three categories represented by up-regulated proteins. Only four down-regulated proteins were in these groups, while most (11/13) down-regulated proteins were categorized under oxidoreductase activity, metal ion binding, and nucleotide binding.

**Fig 4:**
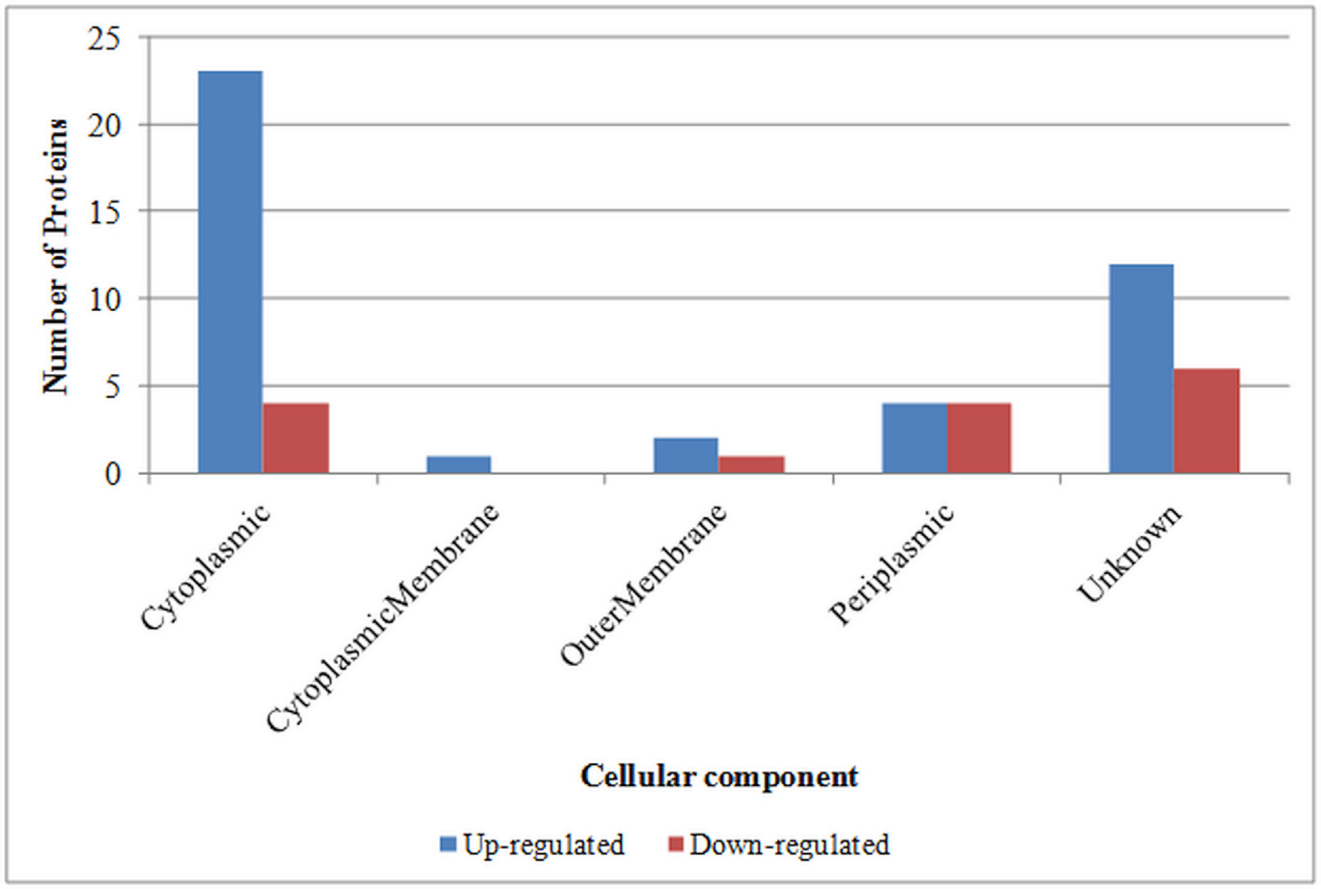
Subcellular locations of *Edwardsiella ictaluri* proteins differentially regulated due to *in vitro* iron-restriction were predicted using PSORTb. Number of differentially abundant proteins, identified in this study, predicted to be located in various subcellular locations was shown. Unknown category includes proteins with multiple subcellular localizations or unknown location.

Subcellular locations of differentially abundant proteins were predicted using PSORTb (Fig 4). Higher numbers of up- and down-regulated proteins were predicted to be located in the cytoplasm and periplasm, excluding those proteins of unknown location.

**Fig 5:**
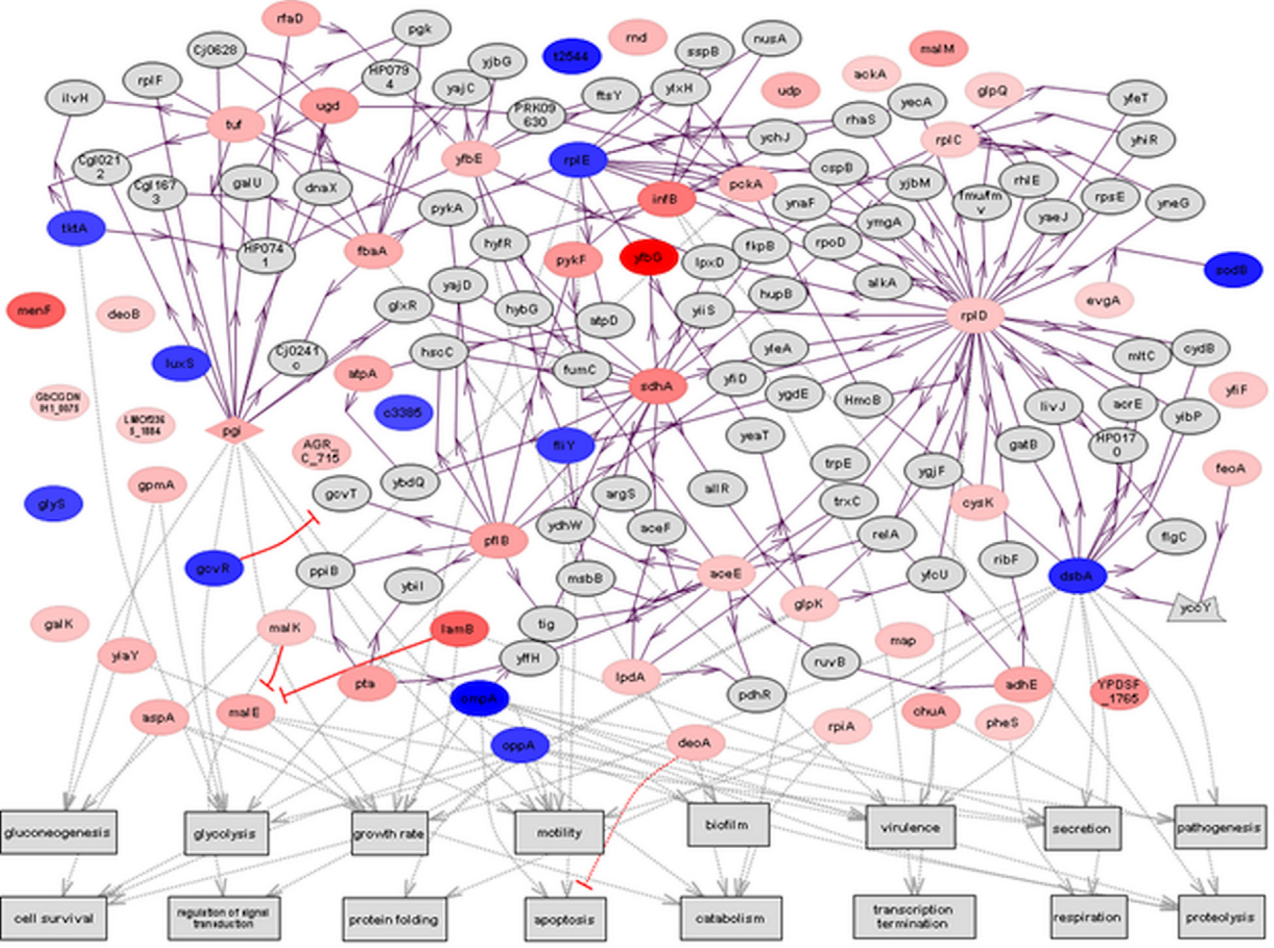
Protein interaction network of differentially regulated *Edwardsiella ictaluri* proteins due to *in vitro* iron-restriction. Entities shown in red and blue were up- and down-regulated protein, respectively, due to *in vitro* iron-restriction. Intensity of color indicates the fold-difference in protein abundance. Each entity represents protein, arrow indicates binding, > indicates post-translational regulation, and → indicates abundance regulation.

Pathways with significant representation of differentially abundant proteins were determined (*p* < 0.05). Ten pathways related to carbohydrate, amino acid, lipid, and nucleotide metabolism were significantly represented (Table 2). We used a pathway reconstruction algorithm, “Build Pathway” available in Pathway Studio, to analyze the shortest paths of up- and down-regulated proteins with biological interactions such as binding interactions, post-translational regulation, and abundance regulation. Cellular processes such as pathogenesis, virulence, secretion, biofilm, motility, regulation of signal transduction, protein folding, glycolysis, gluconeogenesis, growth rate, catabolism, transcription termination, respiration, proteolysis, apoptosis, and cell survival were predominantly represented in the differential protein abundance interaction network (Fig 5).

**Table 2:** List of pathways significantly represented by differentially regulated *Edwardsiella ictaluri* proteins in response to *in vitro* iron-restriction.

Name	No. of proteins	p-value	Classification
Glycolysis / Gluconeogenesis	8	1.29E-06	Carbohydrate Metabolism
Pyruvate metabolism	8	2.31E-06	Carbohydrate Metabolism
Pentose phosphate pathway	5	2.66E-04	Carbohydrate Metabolism
Citrate cycle (TCA cycle)	3	1.06E-02	Carbohydrate Metabolism
Butanoate metabolism	4	1.51E-02	Carbohydrate Metabolism
Propanoate metabolism	3	4.01E-02	Carbohydrate Metabolism
Glycerolipid metabolism	3	1.96E-02	Lipid Metabolism
Selenoamino acid metabolism	6	5.49E-05	Metabolism of Other Amino Acids
Taurine and hypotaurine metabolism	2	4.57E-02	Metabolism of Other Amino Acids
Purine metabolism	5	2.14E-02	Nucleotide Metabolism

## Discussion

The purpose of the present study was to identify differential abundance in proteins of *E*. *ictaluri* grown under iron-restricted and normal growth conditions and investigate their possible role in pathogenesis. We identified 50 unique *E*. *ictaluri* proteins with altered abundance (37 up- and 13 down-regulated) in response to iron-restriction. It is known that iron is an essential micronutrient that acts as a cofactor for enzymes involved in oxidative and electron transport processes. Hence, iron is essential for pathogenic bacteria to establish an infection.

Iron uptake in bacteria is controlled tightly by the ferric uptake regulator (*fur*) gene [28, 50], and it has been shown that the *E*. *ictaluri fur* gene has a similar regulatory function [31]. Transport proteins, especially cation transporters, are highly expressed in iron-restricted conditions. TonB-dependent heme/hemoglobin receptor family protein, with its 18-fold higher abundance in iron-restricted growth conditions, may act as a crucial factor in iron uptake as part of the *E*. *ictaluri hemPRSTUV* operon. Recently, an up-regulation of the *E*. *ictaluri* TonB-dependent heme/hemoglobin receptor in iron limited conditions has also been reported [31]. TonB-dependent heme/hemoglobin receptor family protein in *E*. *ictaluri* might have both receptor and transporter activity along with its involvement in transduction of environmental signals, and a possible role in pathogenicity similar to several bacterial pathogens [31, 51, 52]. Research in *Vibrio alginolyticus* demonstrated that mutants of TonB complex exhibited attenuation in virulence compared to wild-type in zebrafish (*Danio rerio*) [53]. Similarly, maltoporin (LamB), a member of the sugar porin family, aid in transport of maltose and other maltodextrins across the outer membrane in *E*. *coli* [54]. Mutational studies of the *lamB* gene of enteropathogenic *E*. *coli* showed that mutants were deficient in adherence to HEp-2 cells [55]. It was also shown that LamB is an important outer membrane protein in *E*. *coli* for obtaining tetracycline resistance [56]. Based on our understanding, proteins involved in the acquisition of iron are closely associated with virulence of several bacteria; it is likely that transporter proteins identified in the present study might be important in *E*. *ictaluri* pathogenesis.

Translational proteins like elongation factor Tu (EF-Tu), a three-domain GTPase, is crucial during the elongation phase of mRNA translation. The EF-Tu in complex with GTP and aminoacyl-tRNA delivers tRNA to the ribosome. It is known that EF-Tu might play a role in protein-folding during stress [57]. It is also proposed that EF-Tu might sense and respond to stress [58]. Hence, EF-Tu may assume the role of translational regulation allowing it to trigger the synthesis of stress-induced proteins and to thwart the translation of unnecessary proteins. During starvation/stress in *E*. *coli*, EF-Tu was shown to be methylated and become membrane associated [59]. Previous research has also shown that EF-Tu might act as a virulence factor in *P*. *aeruginosa* [60]. During those conditions EF-Tu may play a possible role in the organism’s response to stress and growth regulation, in addition to its primary role in regulation of translation.

N-acetylmuramoyl-L-alanine amidase is an outer membrane lipoprotein which catalyzes cleavage of the bond between muramic acid and L-alanine of murein. Similarly, ADP-L-glycero-D-mannoheptose-6-epimerase is involved in the lipopolysaccharide (LPS) biosynthesis pathway and is responsible for synthesis of the ADP-heptose precursor of core LPS [61]. Glucose-6-phosphate isomerase, phosphoenolpyruvate carboxykinase, hypothetical protein NT01EI_2846, which is also named as Phosphoglyceromutase (GpmA), and Fructose 1, 6-bisphosphate aldolase (FbA) are known to be involved in the glycolysis/gluconeogenesis pathway. Vassinova and Kozyrev, (2000) [62] suggested that transcription of *gpmA* is regulated by Fur in *E*. *coli*. It has also been shown that FbA of *E*. *ictaluri* has antigenic properties and is regulated during the infectious process of ESC in catfish [63]. Similarly, research conducted by Ling et al. (2004) [64] also revealed that on respiratory challenge with virulent *Streptococcus pneumonia* in mice, FbA is able to elicit significant levels of immune response. Succinate dehydrogenase catalyzes the oxidation of succinate to fumerate in the tricarboxylic acid cycle. Aspartate ammonia-lyase is an anerobic enzyme which catalyzes the amination of fumarate to generate L-aspartate. Mutational studies conducted by Jacobsen et al. (2005) [65] showed that aspartate ammonia-lyase plays an important role in the pathogenesis of *Actinobacillus pleuropneumoniae* in pigs [65].

Research conducted by Wang et al. (2009) [66] confirmed that the conserved hypothetical protein (EsrB) and iron concentrations regulate the *E*. *tarda* virulence proteins. Thiol: disulfide interchange protein DsbA, which was -3.35 fold down-regulated, was a protein-folding catalyst which aids in correct folding of surface-presented virulence factors like adherence factors, toxins, and components of the type III secretory system [67]. The observed down-regulation of periplasmic oligopeptide-binding protein (oppA), an ATP-dependent ABC Superfamily of transporters involved in oligopeptide uptake, is in agreement with reduced metabolism of bacteria due to stress caused by the limitation of available iron [68]. Research conducted by Lee et al. (2009) [69] revealed that the *dsbA* mutant of *Pseudomonas putida* exhibited enhanced extracellular matrix production and biofilm formation. Down-regulation of OmpA (4.79 fold), is consistent with findings of *Clamydia pneumonia* an iron-limitation model [70]. Similarly, superoxide dismutase (Sod_Fe) was shown to be positively regulated by the Fur—Fe^+^ complex in many bacterial species [71, 72]. Hence in the present study, down regulation of superoxide dismutase was expected due to growth of *E*. *ictaluri* in iron-restricted conditions [73].

GO annotation and manual slimming of up-regulated proteins resulted in a higher number of biological processes (12) and molecular functional categories (14) compared to down-regulated proteins (7 and 9, respectively). This was expected as the number of unique proteins that were up-regulated (37) was high compared to those that were down-regulated (13). Up-regulated proteins were highly represented in the carbohydrate metabolic process. This might be due to a higher abundance of proteins involved in glycolysis/gluconeogenesis, pyruvate metabolism, and in synthesis of cell wall structures like peptidoglycan and LPS. It has been shown that, bacteria in general, alter their metabolic activity particularly increase their ability to metabolize variety of carbon sources followed by changes in expression pattern of the virulence factors [74, 75]. Similarly, during iron scarce situation, iron-dependent pathways in microbes are diminished while iron-independent enzymes and metabolic pathways are enhanced [76]. Down-regulation of few proteins involved in cellular metabolic processes, oxidation reduction, and translational processes could also indicate a possible reduction in the metabolism of *E*. *ictaluri* due to iron starvation stress.

A higher number of up-regulated proteins were predicted to be located in cytoplasm as they are hydrophilic and thus do not interfere in 2-DE separation techniques. DsbA, Sod_Fe, periplasmic oligopeptide-binding protein, and conserved hypothetical protein were the four down-regulated proteins predicted to be located in periplasm.

Protein interaction networks of differentially abundant proteins were built using Pathway Studio. Differentially abundant proteins were involved in cellular processes like virulence, pathogenesis, secretion, biofilm, and regulation of signal transduction. Up-regulated proteins like FbaA and ChuA and down-regulated proteins like OppA, OmpA, and DsbA were involved in virulence and pathogenesis processes suggesting that differentially abundant proteins during iron-restriction may play an important role in *E*. *ictaluri* pathogenesis. Down-regulation of several proteins involved in cellular processes like cell survival, motility, and growth rate may be expected with reduced metabolism due to iron-limitation stress. Furthermore, up-regulation of proteins involved in carbohydrate metabolism, nucleoside/nucleotide metabolism, TCA cycle, and transport process might be due to a part of global iron homeostatic response of *E*. *ictaluri* replacing iron dependent enzymes with iron-independent alternatives as exhibited by *E*. *coli* and many other bacteria [77–79]. Few proteins involved in biosynthesis process, oxidation reduction, translation, and other processes are down regulated. This might be due to *E*. *ictaluri*’s engagement in an iron-sparing process, similar to *E*. *coli*, to conserve its limited iron resources [80, 81]

It is always a challenge to elucidate how bacteria employs various adaptive mechanisms to invade, colonize, and successfully establish a disease in the host. It is likely that *E*. *ictaluri* will encounter several environmental stresses in the gastric environment of catfish like iron starvation and fluctuation in pH during the initial course of its pathogenesis. With an objective to thoroughly elucidate the response of *E*. *ictaluri* to iron-restriction, we used 2D-DIGE technology to investigate changes protein abundance. We therefore hypothesized that analysis of *E*. *ictaluri* response towards iron-restriction conditions may aid in enlightening the possible mechanisms of pathogenesis of *E*. *ictaluri*. In the present study, we noted several differentially abundant proteins that were previously shown to be involved in the pathogenesis of other Gram-negative bacteria including *E*. *ictaluri*. Future experiments determining the role of these differentially abundant proteins should provide important information regarding the mechanisms used by *E*. *ictaluri* during colonization and establishment of ESC in catfish.

## References

[1] Hawke JP, McWhorter AC, Steigerwalt AG, Brenner DJ. *Edwardsiella ictaluri* sp. nov., the causative agent of enteric septicemia of catfish. Int J Syst Bacteriol. 1981;31:396–400.

[2] Miyazaki T, Plumb JA. Histopathology of *Edwardsiella ictaluri* in channel catfish *Ictalurus punctatus* (Rafinesque). J Fish Dis. 1985;8:389–92.

[3] Newton JC, Wolfe LG, Grizzle JM, Plumb JA. Pathology of experimental enteric septicaemia in channel catfish, *Ictalurus punctatus* (Rafinesque), following immersion-exposure to *Edwardsiella ictaluri* . J Fish Dis. 1989;12:335–47.

[4] Shotts EB, Blazer VS, Waltman WD. Pathogenesis of experimental *Edwardsiella ictaluri* infections in channel catfish (*Ictalurus punctatus*). Can J Fish Aquat Sci. 1986;43(1):36–42.

[5] Stanley LA, Hudson JS, Schwedler TE, Hayasaka SS. Extracellular products associated with virulent and avirulent strains of *Edwardsiella ictaluri* from channel catfish. J Aquat Anim Health. 1994;6(1):36–43.

[6] Lawrence ML, Banes MM, Azadi P, Reeks BY. The *Edwardsiella ictaluri* O polysaccharide biosynthesis gene cluster and the role of O polysaccharide in resistance to normal catfish serum and catfish neutrophils. Microbiology. 2003;149(Pt 6):1409–21. . 12777482 10.1099/mic.0.26138-0

[7] Lawrence ML, Banes MM, Williams ML. Phenotype and virulence of a transposon-derived lipopolysaccharide O side-chain mutant strain of *Edwardsiella ictaluri* . J Aquat Anim Health. 2001;13(4):291–9. ISI:000172799700001.

[8] Arias CR, Shoemaker CA, Evans JJ, Klesius PH. A comparative study of *Edwardsiella ictaluri* parent (EILO) and *E*. *ictaluri* rifampicin-mutant (RE-33) isolates using lipopolysaccharides, outer membrane proteins, fatty acids, Biolog, API 20E and genomic analyses. J Fish Dis. 2003;26(7):415–21. . 12946011 10.1046/j.1365-2761.2003.00475.x

[9] Weete JD, Blevins WT, Chitrakorn S, Saeed MO, Plumb JA. Chemical characterization of lipopolysaccharide from *Edwardsiella ictaluri*, a fish pathogen. Can J Microbiol. 1988;34(11):1224–9. . 3208199 10.1139/m88-215

[10] Newton JC, Triche PL. Electrophoretic and immunochemical characterization of lipopolysaccharide of *Edwardsiella ictaluri* from channel catfish. J Aquat Anim Health. 1993;5(4):246–53.

[11] Williams ML, Azadi P, Lawrence ML. Comparison of cellular and extracellular products expressed by virulent and attenuated strains of *Edwardsiella ictaluri* . J Aquat Anim Health. 2003;15(4):264–73. ISI:000223007700002.

[12] Newton JC, Blevins WT, Wilt GR, Wolfe LG. Outer membrane protein profiles of *Edwardsiella ictaluri* from fish. Am J Vet Res. 1990;51(2):211–5. . 1689127

[13] Skirpstunas RT, Baldwin TJ. Antibodies against affinity-purified, surface-exposed outer membrane proteins of *Edwardsiella ictaluri* block invasion into fathead minnow epithelial cells. J Aquat Anim Health. 2003;15(1):92–7. ISI:000183284200011.

[14] Vinitnantharat S, Plumb JA, Brown AE. Isolation and purification of an outer membrane protein of *Edwardsiella ictaluri* and its antigenicity to channel catfish (*Ictalurus punctatus*). Fish Shellfish Immunol. 1993;3:401–9.

[15] Bader JA, Shoemaker CA, Klesius PH. Immune response induced by N-lauroylsarcosine extracted outer-membrane proteins of an isolate of *Edwardsiella ictaluri* in channel catfish. Fish Shellfish Immunol. 2004;16(3):415–28. . 15123308 10.1016/j.fsi.2003.07.003

[16] Williams ML, Lawrence ML. Identification and characterization of a two-component hemolysin from *Edwardsiella ictaluri* . Vet Microbiol. 2005;108(3–4):281–9. . 15923091 10.1016/j.vetmic.2005.04.017

[17] Cooper RK, Shotts EB, Nolan LK. Use of a minitransposon to study chondroitinase activity associated with *Edwardsiella ictaluri* . J Aquat Anim Health. 1996;8:319–24.

[18] Waltman WD, Shotts EB, Hsu TC. Biochemical characteristics of Edwardsiella ictaluri. Appl Environ Microbiol. 1986;51(1):101–4. Epub 1986/01/01. . 3954336 10.1128/aem.51.1.101-104.1986 PMC238823

[19] Ainsworth AJ, Chen DX. Differences in the phagocytosis of four bacteria by channel catfish neutrophils. Dev Comp Immunol. 1990;14(2):201–9. Epub 1990/01/01. . 2369968 10.1016/0145-305x(90)90091-r

[20] Baldwin TJ, Newton JC. Pathogenesis of enteric septicemia of channel catfish, caused by *Edwardsiella ictaluri*: bacteriologic and light and electron microscopic findings. J Aquat Anim Health. 1993;5:189–98.

[21] Mey AR, Wyckoff EE, Kanukurthy V, Fisher CR, Payne SM. Iron and fur regulation in Vibrio cholerae and the role of fur in virulence. Infect Immun. 2005;73(12):8167–78. Epub 2005/11/22. 73/12/8167 [pii] 10.1128/IAI.73.12.8167-8178.2005 . 16299312 PMC1307094

[22] Masse E, Arguin M. Ironing out the problem: new mechanisms of iron homeostasis. Trends in biochemical sciences. 2005;30(8):462–8. 10.1016/j.tibs.2005.06.005 . 15996868

[23] Ratledge C, Dover LG. Iron metabolism in pathogenic bacteria. Annu Rev Microbiol. 2000;54:881–941. Epub 2000/10/06. [pii]. . 11018148 10.1146/annurev.micro.54.1.881

[24] Lenco J, Hubalek M, Larsson P, Fucikova A, Brychta M, Macela A, et al Proteomics analysis of the Francisella tularensis LVS response to iron restriction: induction of the F. tularensis pathogenicity island proteins IglABC. FEMS Microbiol Lett. 2007;269(1):11–21. Epub 2007/01/18. FML595 [pii] 10.1111/j.1574-6968.2006.00595.x . 17227466

[25] Payne SM. Iron acquisition in microbial pathogenesis. Trends Microbiol. 1993;1(2):66–9. Epub 1993/05/01. . 8044465 10.1016/0966-842x(93)90036-q

[26] Weinberg ED. The development of awareness of iron-withholding defense. Perspect Biol Med. 1993;36(2):215–21. Epub 1993/01/01. . 8446492 10.1353/pbm.1993.0063

[27] Litwin CM, Calderwood SB. Role of iron in regulation of virulence genes. Clin Microbiol Rev. 1993;6(2):137–49. Epub 1993/04/01. . 8472246 10.1128/cmr.6.2.137 PMC358274

[28] Griffiths E, Chart H. Iron as a regulatory signal in Iron and Infection. 2 ed BJaG E, editor: Wiley, John & Sons; 1999 5 1999. 526 p.

[29] Bindereif A, Neilands JB. Promoter mapping and transcriptional regulation of the iron assimilation system of plasmid ColV-K30 in Escherichia coli K-12. J Bacteriol. 1985;162(3):1039–46. Epub 1985/06/01. . 2581932 10.1128/jb.162.3.1039-1046.1985 PMC215880

[30] Calderwood SB, Mekalanos JJ. Iron regulation of Shiga-like toxin expression in Escherichia coli is mediated by the fur locus. J Bacteriol. 1987;169(10):4759–64. Epub 1987/10/01. . 3308853 10.1128/jb.169.10.4759-4764.1987 PMC213851

[31] Santander J, Golden G, Wanda SY, Curtiss R 3rd. Fur-regulated iron uptake system of Edwardsiella ictaluri and its influence on pathogenesis and immunogenicity in the catfish host. Infect Immun. 2012;80(8):2689–703. 10.1128/IAI.00013-12 22615248 PMC3434582

[32] Dubos RJ, Geiger JW. Preparation and Properties of Shiga Toxin and Toxoid. J Exp Med. 1946;84(2):143–56. Epub 1946/07/31. . 19871559 PMC2135652

[33] Goldberg MB, DiRita VJ, Calderwood SB. Identification of an iron-regulated virulence determinant in Vibrio cholerae, using TnphoA mutagenesis. Infect Immun. 1990;58(1):55–60. Epub 1990/01/01. . 2152889 10.1128/iai.58.1.55-60.1990 PMC258408

[34] Sciortino CV, Finkelstein RA. Vibrio cholerae expresses iron-regulated outer membrane proteins in vivo. Infect Immun. 1983;42(3):990–6. Epub 1983/12/01. . 6642675 10.1128/iai.42.3.990-996.1983 PMC264397

[35] Sigel SP, Payne SM. Effect of iron limitation on growth, siderophore production, and expression of outer membrane proteins of Vibrio cholerae. J Bacteriol. 1982;150(1):148–55. Epub 1982/04/01. . 6460753 10.1128/jb.150.1.148-155.1982 PMC220093

[36] Stoebner JA, Payne SM. Iron-regulated hemolysin production and utilization of heme and hemoglobin by Vibrio cholerae. Infect Immun. 1988;56(11):2891–5. Epub 1988/11/01. . 2971620 10.1128/iai.56.11.2891-2895.1988 PMC259667

[37] Dyer DW, West EP, McKenna W, Thompson SA, Sparling PF. A pleiotropic iron-uptake mutant of Neisseria meningitidis lacks a 70-kilodalton iron-regulated protein. Infect Immun. 1988;56(4):977–83. Epub 1988/04/01. . 3126152 10.1128/iai.56.4.977-983.1988 PMC259401

[38] Bjorn MJ, Iglewski BH, Ives SK, Sadoff JC, Vasil ML. Effect of iron on yields of exotoxin A in cultures of Pseudomonas aeruginosa PA-103. Infect Immun. 1978;19(3):785–91. Epub 1978/03/01. . 417030 10.1128/iai.19.3.785-791.1978 PMC422257

[39] Bjorn MJ, Sokol PA, Iglewski BH. Influence of iron on yields of extracellular products in Pseudomonas aeruginosa cultures. J Bacteriol. 1979;138(1):193–200. Epub 1979/04/01. . 108250 10.1128/jb.138.1.193-200.1979 PMC218257

[40] Poole K, Neshat S, Krebes K, Heinrichs DE. Cloning and nucleotide sequence analysis of the ferripyoverdine receptor gene fpvA of Pseudomonas aeruginosa. J Bacteriol. 1993;175(15):4597–604. Epub 1993/08/01. . 8335619 10.1128/jb.175.15.4597-4604.1993 PMC204910

[41] Dumpala PR, Lawrence ML, Karsi A. Proteome analysis of Edwardsiella ictaluri. Proteomics. 2009;9(5):1353–63. Epub 2009/03/03. 10.1002/pmic.200800652 . 19253294

[42] Lawrence ML, Cooper RK, Thune RL. Attenuation, persistence, and vaccine potential of an Edwardsiella ictaluri purA mutant. Infect Immun. 1997;65(11):4642–51. 9353045 10.1128/iai.65.11.4642-4651.1997 PMC175666

[43] Davies RL, Parton R, Coote JG, Gibbs HA, Freer JH. Outer-membrane protein and lipopolysaccharide variation in Pasteurella haemolytica serotype A1 under different growth conditions. J Gen Microbiol. 1992;138(5):909–22. Epub 1992/05/01. 1645128 10.1099/00221287-138-5-909

[44] Sabri M, Leveille S, Dozois CM. A SitABCD homologue from an avian pathogenic Escherichia coli strain mediates transport of iron and manganese and resistance to hydrogen peroxide. Microbiology. 2006;152(Pt 3):745–58. Epub 2006/03/04. 152/3/745 [pii] 10.1099/mic.0.28682-0 . 16514154

[45] Abdelhamed H, Lu J, Shaheen A, Abbass A, Lawrence ML, Karsi A. Construction and evaluation of an Edwardsiella ictaluri fhuC mutant. Vet Microbiol. 2013;162(2–4):858–65. 10.1016/j.vetmic.2012.11.006 . 23201245

[46] Chaudhary A, Pechan T, Willett KL. Differential protein expression of peroxiredoxin I and II by benzo(a)pyrene and quercetin treatment in 22Rv1 and PrEC prostate cell lines. Toxicol Appl Pharmacol. 2007;220(2):197–210. Epub 2007/02/13. S0041-008X(06)00505-9 [pii] 10.1016/j.taap.2006.12.030 . 17292933

[47] McCarthy FM, Wang N, Magee GB, Nanduri B, Lawrence ML, Camon EB, et al AgBase: a functional genomics resource for agriculture. BMC Genomics. 2006;7:229 Epub 2006/09/12. 1471-2164-7-229 [pii] 10.1186/1471-2164-7-229 . 16961921 PMC1618847

[48] Binns D, Dimmer E, Huntley R, Barrell D, O'Donovan C, Apweiler R. QuickGO: a web-based tool for Gene Ontology searching. Bioinformatics. 2009;25(22):3045–6. Epub 2009/09/12. btp536 [pii] 10.1093/bioinformatics/btp536 . 19744993 PMC2773257

[49] Gardy JL, Laird MR, Chen F, Rey S, Walsh CJ, Ester M, et al PSORTb v.2.0: expanded prediction of bacterial protein subcellular localization and insights gained from comparative proteome analysis. Bioinformatics. 2005;21(5):617–23. Epub 2004/10/27. bti057 [pii] 10.1093/bioinformatics/bti057 . 15501914

[50] Braun V. Iron uptake mechanisms and their regulation in pathogenic bacteria. Int J Med Microbiol. 2001;291(2):67–79. Epub 2001/07/05. . 11437341 10.1078/1438-4221-00103

[51] Ferguson AD, Amezcua CA, Halabi NM, Chelliah Y, Rosen MK, Ranganathan R, et al Signal transduction pathway of TonB-dependent transporters. Proc Natl Acad Sci U S A. 2007;104(2):513–8. Epub 2007/01/02. 0609887104 [pii] 10.1073/pnas.0609887104 . 17197416 PMC1760641

[52] Koebnik R. TonB-dependent trans-envelope signalling: the exception or the rule? Trends Microbiol. 2005;13(8):343–7. Epub 2005/07/05. S0966-842X(05)00163-0 [pii] 10.1016/j.tim.2005.06.005 . 15993072

[53] Wang Q, Liu Q, Cao X, Yang M, Zhang Y. Characterization of two TonB systems in marine fish pathogen Vibrio alginolyticus: their roles in iron utilization and virulence. Arch Microbiol. 2008;190(5):595–603. Epub 2008/07/17. 10.1007/s00203-008-0407-1 . 18629473

[54] Wang YF, Dutzler R, Rizkallah PJ, Rosenbusch JP, Schirmer T. Channel specificity: structural basis for sugar discrimination and differential flux rates in maltoporin. J Mol Biol. 1997;272(1):56–63. Epub 1997/09/23. S0022-2836(97)91224-9 [pii] 10.1006/jmbi.1997.1224 . 9299337

[55] Subramanian K, Shankar RB, Meenakshisundaram S, Lakshmi BS, Williams PH, Balakrishnan A. LamB-mediated adherence of enteropathogenic Escherichia coli to HEp-2 cells. J Appl Microbiol. 2008;105(3):715–22. Epub 2008/04/10. JAM3800 [pii] 10.1111/j.1365-2672.2008.03800.x . 18397259

[56] Zhang DF, Jiang B, Xiang ZM, Wang SY. Functional characterisation of altered outer membrane proteins for tetracycline resistance in Escherichia coli. Int J Antimicrob Agents. 2008;32(4):315–9. Epub 2008/07/16. S0924-8579(08)00200-8 [pii] 10.1016/j.ijantimicag.2008.04.015 . 18620846

[57] Caldas TD, El Yaagoubi A, Richarme G. Chaperone properties of bacterial elongation factor EF-Tu. J Biol Chem. 1998;273(19):11478–82. Epub 1998/06/13. . 9565560 10.1074/jbc.273.19.11478

[58] Yu F, Inouye S, Inouye M. Lipoprotein-28, a cytoplasmic membrane lipoprotein from Escherichia coli. Cloning, DNA sequence, and expression of its gene. J Biol Chem. 1986;261(5):2284–8. Epub 1986/02/15. . 3003106

[59] Young CC, Bernlohr RW. Elongation factor Tu is methylated in response to nutrient deprivation in Escherichia coli. J Bacteriol. 1991;173(10):3096–100. Epub 1991/05/01. . 2022614 10.1128/jb.173.10.3096-3100.1991 PMC207902

[60] Kunert A, Losse J, Gruszin C, Huhn M, Kaendler K, Mikkat S, et al Immune evasion of the human pathogen Pseudomonas aeruginosa: elongation factor Tuf is a factor H and plasminogen binding protein. J Immunol. 2007;179(5):2979–88. Epub 2007/08/22. 179/5/2979 [pii]. . 17709513 10.4049/jimmunol.179.5.2979

[61] Kneidinger B, Marolda C, Graninger M, Zamyatina A, McArthur F, Kosma P, et al Biosynthesis pathway of ADP-L-glycero-beta-D-manno-heptose in Escherichia coli. J Bacteriol. 2002;184(2):363–9. Epub 2001/12/26. . 11751812 10.1128/JB.184.2.363-369.2002 PMC139585

[62] Vassinova N, Kozyrev D. A method for direct cloning of fur-regulated genes: identification of seven new fur-regulated loci in Escherichia coli. Microbiology. 2000;146 Pt 12:3171–82. Epub 2000/12/02. . 11101675 10.1099/00221287-146-12-3171

[63] Moore MM, Fernandez DL, Thune RL. Cloning and characterization of *Edwardsiella ictaluri* proteins expressed and recognized by the channel catfish, *Ictalurus punctatus*, immune response during infection. Dis Aquat Organ. 2002;52(2):93–107. . 12542086 10.3354/dao052093

[64] Ling E, Feldman G, Portnoi M, Dagan R, Overweg K, Mulholland F, et al Glycolytic enzymes associated with the cell surface of Streptococcus pneumoniae are antigenic in humans and elicit protective immune responses in the mouse. Clin Exp Immunol. 2004;138(2):290–8. Epub 2004/10/23. CEI2628 [pii] 10.1111/j.1365-2249.2004.02628.x . 15498039 PMC1809218

[65] Jacobsen I, Hennig-Pauka I, Baltes N, Trost M, Gerlach GF. Enzymes involved in anaerobic respiration appear to play a role in Actinobacillus pleuropneumoniae virulence. Infect Immun. 2005;73(1):226–34. Epub 2004/12/25. 73/1/226 [pii] 10.1128/IAI.73.1.226-234.2005 . 15618158 PMC538954

[66] Wang X, Wang Q, Xiao J, Liu Q, Wu H, Xu L, et al Edwardsiella tarda T6SS component evpP is regulated by esrB and iron, and plays essential roles in the invasion of fish. Fish Shellfish Immunol. 2009;27(3):469–77. Epub 2009/07/01. S1050-4648(09)00209-5 [pii] 10.1016/j.fsi.2009.06.013 . 19563898

[67] Yu J, Kroll JS. DsbA: a protein-folding catalyst contributing to bacterial virulence. Microbes Infect. 1999;1(14):1221–8. Epub 1999/12/03. S1286-4579(99)00239-7 [pii]. . 10580278 10.1016/s1286-4579(99)00239-7

[68] Madsen ML, Nettleton D, Thacker EL, Minion FC. Transcriptional profiling of Mycoplasma hyopneumoniae during iron depletion using microarrays. Microbiology. 2006;152(Pt 4):937–44. Epub 2006/03/22. 152/4/937 [pii] . 16549658 10.1099/mic.0.28674-0

[69] Lee Y, Oh S, Park W. Inactivation of the Pseudomonas putida KT2440 dsbA gene promotes extracellular matrix production and biofilm formation. FEMS Microbiol Lett. 2009;297(1):38–48. Epub 2009/06/09. FML1650 [pii] 10.1111/j.1574-6968.2009.01650.x . 19500143

[70] Timms P, Good D, Wan C, Theodoropoulos C, Mukhopadhyay S, Summersgill J, et al Differential transcriptional responses between the interferon-gamma-induction and iron-limitation models of persistence for Chlamydia pneumoniae. J Microbiol Immunol Infect. 2009;42(1):27–37. Epub 2009/05/09. . 19424556

[71] Jung YS, Kwon YM. Small RNA ArrF regulates the expression of sodB and feSII genes in Azotobacter vinelandii. Curr Microbiol. 2008;57(6):593–7. Epub 2008/10/03. 10.1007/s00284-008-9248-z . 18830664

[72] Vasil ML. How we learnt about iron acquisition in Pseudomonas aeruginosa: a series of very fortunate events. Biometals. 2007;20(3–4):587–601. Epub 2006/12/23. 10.1007/s10534-006-9067-2 . 17186376

[73] Ernst FD, Homuth G, Stoof J, Mader U, Waidner B, Kuipers EJ, et al Iron-responsive regulation of the Helicobacter pylori iron-cofactored superoxide dismutase SodB is mediated by Fur. J Bacteriol. 2005;187(11):3687–92. 10.1128/JB.187.11.3687-3692.2005 15901691 PMC1112043

[74] Antiabong JF, Ball AS, Brown MH. The effects of iron limitation and cell density on prokaryotic metabolism and gene expression: Excerpts from Fusobacterium necrophorum strain 774 (sheep isolate). Gene. 2015;563(1):94–102. 10.1016/j.gene.2015.03.017 . 25771225

[75] Sheldon JR, Marolda CL, Heinrichs DE. TCA cycle activity in Staphylococcus aureus is essential for iron-regulated synthesis of staphyloferrin A, but not staphyloferrin B: the benefit of a second citrate synthase. Mol Microbiol. 2014;92(4):824–39. 10.1111/mmi.12593 . 24666349

[76] Kaplan J, McVey Ward D, Crisp RJ, Philpott CC. Iron-dependent metabolic remodeling in S. cerevisiae. Biochim Biophys Acta. 2006;1763(7):646–51. 10.1016/j.bbamcr.2006.03.008 . 16697062

[77] Gaballa A, Antelmann H, Aguilar C, Khakh SK, Song KB, Smaldone GT, et al The Bacillus subtilis iron-sparing response is mediated by a Fur-regulated small RNA and three small, basic proteins. Proc Natl Acad Sci U S A. 2008;105(33):11927–32. 10.1073/pnas.0711752105 18697947 PMC2575260

[78] Andrews SC, Robinson AK, Rodriguez-Quinones F. Bacterial iron homeostasis. FEMS Microbiol Rev. 2003;27(2–3):215–37. . 12829269 10.1016/S0168-6445(03)00055-X

[79] Wilderman PJ, Sowa NA, FitzGerald DJ, FitzGerald PC, Gottesman S, Ochsner UA, et al Identification of tandem duplicate regulatory small RNAs in Pseudomonas aeruginosa involved in iron homeostasis. Proc Natl Acad Sci U S A. 2004;101(26):9792–7. 10.1073/pnas.0403423101 15210934 PMC470753

[80] Masse E, Vanderpool CK, Gottesman S. Effect of RyhB small RNA on global iron use in Escherichia coli. J Bacteriol. 2005;187(20):6962–71. 10.1128/JB.187.20.6962-6971.2005 16199566 PMC1251601

[81] McHugh JP, Rodriguez-Quinones F, Abdul-Tehrani H, Svistunenko DA, Poole RK, Cooper CE, et al Global iron-dependent gene regulation in Escherichia coli. A new mechanism for iron homeostasis. J Biol Chem. 2003;278(32):29478–86. 10.1074/jbc.M303381200 . 12746439

